# Learning About Parenting Together: A Programme to Support Parents with Inter-generational Concerns in Pune, India

**DOI:** 10.1007/s10591-017-9429-7

**Published:** 2017-09-18

**Authors:** Emma E. de Wit, Adithy, Cheryl Chakranarayan, Joske F. G. Bunders-Aelen, Barbara J. Regeer

**Affiliations:** 1 Athena Institute for Research on Innovation and Communication in Health and Life Sciences, Faculty of Earth and Life Sciences, VU University, Amsterdam, de Boelelaan 1085, 1081 HV Amsterdam, The Netherlands; 2 Pune, India; 3 Central Michigan University, Mount Pleasant, Michigan USA

**Keywords:** Parenting stress, Intervention, India, Adolescents, Inter-generational stress

## Abstract

Rapid developments in the last few decades have brought about dramatic changes in Indian social life, particularly affecting new middle-class families. Inter-generational conflicts, high academic pressures, and modern anxieties lead to stress both in parents and in children. There is a need for parenting programmes that respond to these specific concerns, in order to reduce parenting stress and improve family well-being. This study aimed to develop and evaluate a parenting programme in Pune, India, based on a ‘theory of change’. In this pilot programme, parents were encouraged to learn in a group format about fostering autonomy in children, promoting academic potential in a stress-free manner and remaining in connection with adolescents. Facilitated by a psychologist, parents participated in four sessions involving ‘creativity and play’, ‘self-awareness and bonding’, and ‘communication’. The intervention was based on validated psychotherapeutic approaches and parenting methods to support parents in their learning. Some 16 in-depth interviews were conducted with parents before and eight interviews after the intervention to understand their learning experiences. Additional feedback was gathered from observation notes and debriefings after each session. The results show that the attention for playful quality time helped parents to (re)gain a more creative and flexible attitude towards spending time with their children. Second, parents learned to reflect on their frameworks of meaning (rooted in their own upbringing), listen attentively and communicate more mindful with their children. Furthermore, this study shows that an activity-based approach, connecting parents in co-creating new parenting paradigms, while supporting them with stress-reducing tools, is a useful way of engaging this target group. The study concludes by offering new perspectives for counsellors working with families in countries undergoing rapid change.

## Introduction

Parents clearly play an important role in fostering their children’s well-being. Safe and supportive home environments, and positive parenting styles, are generally considered to have a protective influence on children’s mental health (Bögels and Brechman-Toussaint 2006). In contexts such as urban India, where children and young adults are increasingly dealing with psycho-social issues such as substance abuse, insecurity, stress and anxiety, and sometimes suicidal tendencies (Arun and Chavan 2009; Radhakrishnan et al. 2016; Patel et al. 2012), protective home environments may be crucial (Mitra et al. 2012; Priti and Chauhan 2009; Menon 2013; Verma et al. 2002). At the same time, parenting is likely to be experienced as more complex, counter-intuitive and taxing in countries, such as India, which are undergoing rapid societal transitions (Chadda and Deb 2013; Natrajan and Thomas 2002).

Parenting is perceived as increasingly challenging for families living in modern environments with higher workloads, reduced social support and greater daily stresses (Long 2004). Results from a study in 16 industrialized countries, conducted by Gauthier et al. (2004), show that parents continue to spend more time on parenting, despite the fact they often also work more. In India, for example, the number of two-income couples increased by 58% between 1979 and 1996 (Census of India 2001). Similarly, societal norms increasingly emphasize parents’ obligation to be actively involved in every aspect of their children’s social and psycho-educational development (Furedi 2001), putting pressure on parents and also on children, who experience increasing parental intentions and control (Lee et al. 2010; Natrajan and Thomas 2002). Subject to these norms, parents are intensively involved in their children’s education, closely monitoring their food intake, school bags, teachers, homework projects, and results (Lee et al. 2010; Deb et al. 2013), while also worrying about protecting them from harmful practices on the internet and monitoring their relations with peers (Manjikian 2012, p. 9). For instance, Natrajan and Thomas (2002, p. 491) describe how middle-class parents in Madras worry about their children receiving too much information, exposing them to more ideas than when they were young, making it harder for them to ‘keep up’.

In India, parents appear to be putting pressure on their children to do well academically, comparing grades and emphasizing the importance of exams and career paths (Deb et al. 2010, 2015; Natrajan and Thomas 2002), filling children’s ‘free time’ with extra tuition^1^ (Sharma 2007). An additional problem is that children receive different information than their parents and hold more Western notions about how to live and express their individuality. This can lead to parent–child conflicts, particularly when parents place high values on family relationships and patriarchal control (Albert et al. 2007). Carson and Chowdhury (2000) note that conflicts often occur when parents insist on monitoring their children’s social behaviour while, at the same time, modern adolescents are continuing to seek out new ways to meet up with peers, experimenting with relationships and sexual intimacy. The concept of inter-generational conflicts in India was described back in 1970 by Gangrade (1970, p. 925), as: ‘the difference, gap, distance or conflict of values between the adult and the adolescent generations.’ It seems that countries like India are encountering acculturation problems similar to those in families that migrate from one culture to another (Chadda and Deb 2013; Fitzpatrick and Garcia 2016; Butts et al. n.d.), as demonstrated also in cross-cultural studies from the USA and Europe (Farver et al. 2007; Kumar 2002; Baptiste 2005; Ahn et al. 2014). India’s sustained economic development over the past few decades, as well as processes of individualization, urbanization, intensification of mass media use and modern communication, have thus created acculturation challenges for both children and parents (Jiloha 2009; Carson and Chowdhury 2000; Chaudhary and Joseph 2010). This transition has led to a generation of parents who tend to feel lost regarding their role, not knowing when to ‘push’ or when to ‘let go’. It seems that the quickly rising group of ‘middle-class’ Indian parents is especially affected and yet there are too few holistic parenting programmes (Natrajan and Thomas 2002).

Domestic factors also play a role in modern parenting, related to the increasingly blurred image of Indian family life and how household members relate to each other. An increase in the number of nuclear families (couples living alone with their children, without the extended family), and ‘transitional’ households (nuclear families that still live with their extended family but with more autonomy for the parenting couple, for instance, financially), could be causing what Chadda and Deb (2013, p. 301) call ‘temporal compression’:

These changes, which include a shift from joint/extended to nuclear family, along with problems of urbanization, changes of role, status and power with increased employment of women, migratory movements among the younger generation, and loss of the experience advantage of elderly members in the family, have increased the stress and pressure on such families, leading to a greater vulnerability to emotional problems and disorders.

Parenting stress is known to have a negative impact on children’s development. When people are stressed or anxious they are less able to acknowledge the perspective or emotional state of the other, which is necessary for empathic interactions with a child (Johnson et al. 2009; Luthar et al. 2006; Bögels et al. 2010). Indeed, numerous studies reflect on the adverse outcomes of parenting stress on children’s development (Vaughan et al. 2012; Deater-Deckard 1998; Kennedy 2012). These studies show that stress in parents, as an important mediator, should be taken seriously in any parental support programme, particularly in challenging contexts. Patel et al. (2007) argue that young people’s mental health is supported by strengthening the fundamental nurturing qualities in the family, including the well-being of parents who are dealing with inter-generational challenges. Instead of focusing on correcting maladaptive parenting or providing particular parenting advice, scholars advise first working on understanding and then relieving parenting stressors (Kotchick and Forehand 2002; Baptiste 2005; Meyers 1998).

In sum, there is a great need for more mental health services available to middle-class parents in India (Natrajan and Thomas 2002), particularly to address these issues that have been created by rapid socio-economic changes. Hence, in this study we pose the following question: How can a parenting programme help to relieve stress, as well as support parents facing various inter-generational challenges? As such, we explore what parents may have learned from the programme, as well as the parameters that contribute to such learning effects.

### A Creative Stress-Relief Programme for Parents: Developing a Theory of Change

To guide the study in responding to the above question, a ‘Theory of Change’ (TOC) was developed, for which we followed the process described below. In a small team, including a child psychiatrist, a psychologist and two researchers, the core principles for a weekly parenting programme, the *Creative Stress Relief Programme for Parents*, were developed, involving four sessions. The core principles of the programme were chosen as a means to reduce parenting stress (and in turn the stress and anxiety of their children). The content was further developed in response to specific parenting questions that were articulated by the participants in our study [see methodology for further explanation on this Participatory Action Research (PAR) process]. Based on this exploration, three main parenting concerns were identified:

A: How can we, as parents, help our children attain their academic potential, while shielding them from too much societal pressure, and without becoming too stressed or burdening them?

B: How can we remain better connected with our children as they grow up in this new era that is unfamiliar and sometimes daunting to us?

C: How can we help our children to become independent and autonomous individuals without risking physical or mental harm?

The above questions are inherently complex, requiring a flexible, interactive learning approach, as well as research-based principles to bring about change. A TOC was employed as it explains how to work backwards from a desired, communal goal to understanding which actions and principles could contribute to achieving this goal more effectively, in a particular context (Anderson and Kohler 2013, p. 4):

A group of early and intermediate accomplishments sets the stage for producing long-range results. A more complete theory of change articulates the assumptions about the process through which change will occur, and specifies the ways in which all of the required early and intermediate outcomes related to achieving the desired long-term change will be brought about and documented as they occur.

**Fig. 1:**
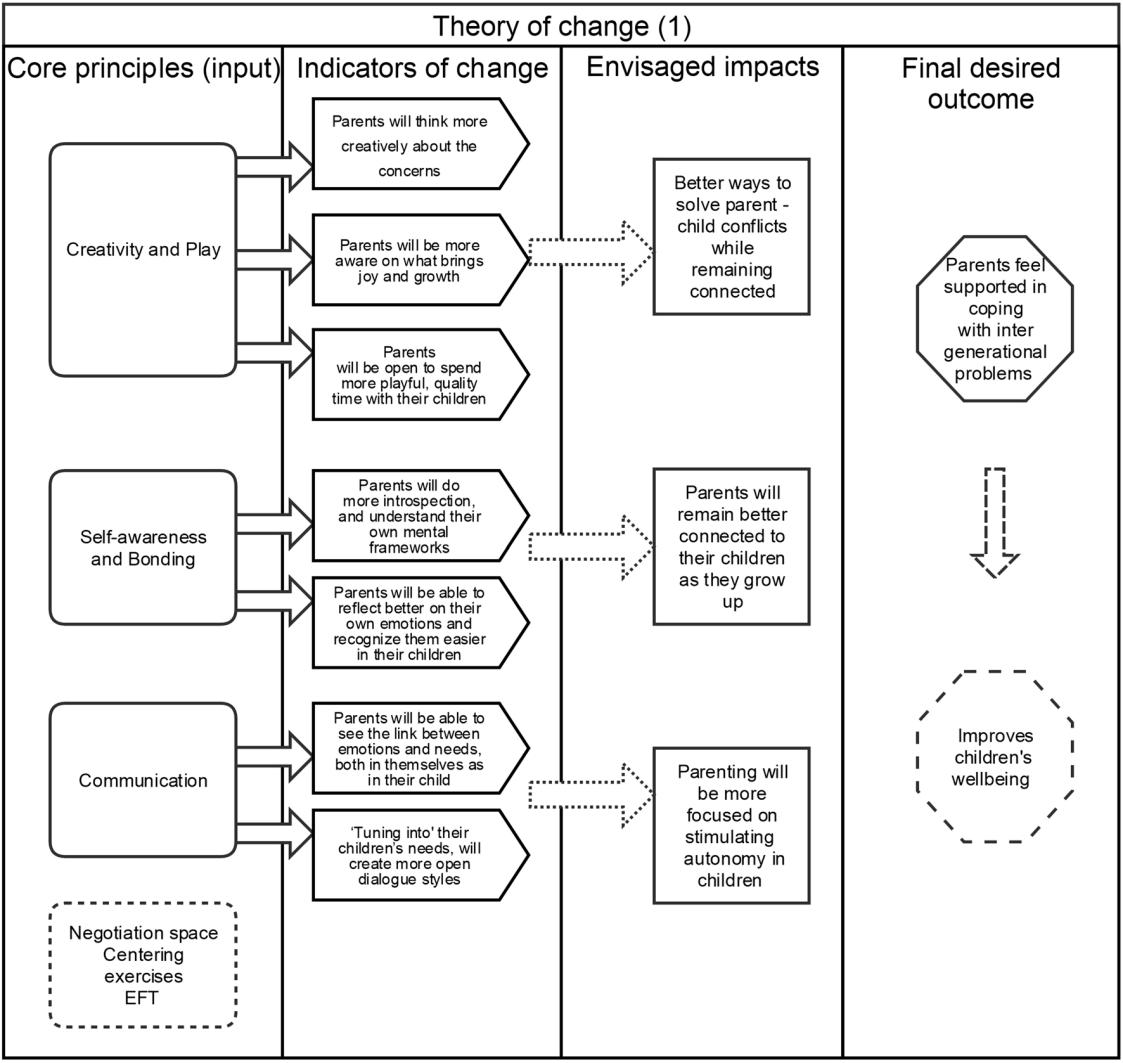
Theory of change for parents in inter-generational contexts

The approach of studying both the impact, as well as the conditions under which benefits are achieved, is particularly useful in India, as studies have shown that there may still be some stigma and resistance towards participating in family therapy programmes, leading to various cultural challenges (Natrajan and Thomas 2002). The TOC, including the core principles, indicators of change, and desired outcomes, is shown below in Fig. 1.

Regarding the core principles of the programme, three important antidotes to stress were considered, namely ‘Creativity and Play’ (session 1), ‘Self-Awareness and Bonding’ (session 2), and ‘Communication’ (sessions 3 and 4). These principles formed the basis of the sessions and many of the group activities, thus stimulating parents to become more playful in their personal habits and expectations, reflective on their own Internal Working Models (IWMs), and to look at important aspects of parent–child communication. The arguments for these principles are presented in Box 1. Additional components of the programme were Body–Mind Centring (BMC) approaches, psycho-education about triggers and stressful responses, as well as Emotional Freedom Technique (EFT). BMC is often used in therapies to help participants ground their awareness in the present (Cohen 2009), and is a technique associated with mindfulness approaches, such as body-scanning or meditation, which can improve the regulation of emotions, bonding and attachment in families (Gambrel and Keeling 2010; Bögels et al. 2010). EFT is a non-invasive ‘meridian’ therapy, which is helpful in stimulating the moment-to-moment processing of emotional responses. EFT ‘tapping’ (using one’s own fingers) is accessible as it can be taught to participants for self-administering on various acupressure points around the body (Boath et al. 2013).

**Table:** 

**Box 1: Core principles of the Creative Stress Relief Programme**
Creativity and Play: Creativity and play are useful principles for three main reasons. First, they allow a sense of joy, aliveness and spontaneity and can therefore help to stimulate a positive and engaging atmosphere within a group. In such a setting, people may find it easier to share their stories and explore difficult emotions (Jennings et al. 1994, pp. 16–20; Richards 2010; Carson and Chowdhury 2000). Second, creativity can be seen as an important antidote to fixed educational expectations that do not necessarily bring joy through learning (as explained by Sir. Ken Robinson in his Ted Talk in 2011). Creative thinking can also promote liberation from pre-defined goals (e.g. ‘I want my child to be an engineer’) often witnessed in Indian middle-class families (Natrajan and Thomas 2002). Finally, creativity is known to stimulate problem-solving and relieve stress (Stuckey and Nobel 2010; Plucker and Dow 2010). Play should be appreciated here as a broader concept than actual ‘game playing’ and as a way to be playful, creative and humorous in everyday situations, including the disciplining and education of children. Stimulating play can also help to improve the quality of interactions between parent and child.
Self-Awareness and Bonding: Parents can find certain interactions with their children difficult (for instance, open dialogues about sexual feelings), which could be due to fears derived from their own childhood and an unresolved attachment state of mind (Lionetti et al. 2015). In attachment theories, these anxieties are often described as rooted in individuals’ IWMs. These cognitive frameworks form the foundation of parents’ expectations of their children and reactions to their behaviour (Kobak and Esposito 2004). In India, this is seen, for instance, where mothers felt they were not given enough freedom of choice and trust as they were growing up, making them more hesitant and less confident towards life in general (Singh and Bhayana 2014). In this programme, parents were invited to explore parent–adolescent bonding, particularly by focusing on their own IWMs and secure attachment narratives (Bowlby 1973). To facilitate part of this session, the psychologist used techniques that are at the basis of trauma-healing theories [such as, for instance, Eye Movement Desensitisation and Reprocessing (EMDR)] to facilitate the exploration of parents’ own thoughts, beliefs and feelings (Shapiro 2001). Negative cognitions such as ‘I’m not good enough’, ‘I’m not safe’ or ‘I am not in control’, rooted in parents’ own upbringing, were analysed together and ‘healed’ by Emotional Freedom Techniques (EFTs).
Communication: Professionals considered communication as a key aspect to be included in the workshop, as inter-generational communication tends to be a notable challenge for middle-class Indian families (Natarjan et al. 2002). The methods used in other parent–child programmes were regarded as useful in stimulating positive parent–adolescent communication, such as derived from Faber and Mazlish (2004), authors of *How to talk so teens will listen, and how to listen so teens will talk*, and *Non-Violent Communication* (NVC) by Marshall Rosenberg (2003). Central to such approaches are the focus on tuning into the child’s emotional state, without aiming to consult or change it directly, and to support the child in communicating their current emotional state (Schrodt et al. 2009). In the group, a combination of such approaches was used to explore fictional as well as real-life situations of parents and children.

The programme was furthermore designed to engage parents intellectually, physically, socially and spiritually, thus creating a holistic balance by combining different exercises. Each session started with some Body–Mind Centring exercises, a warm-up task and reflections on the previous week’s homework. Then the facilitator would continue by providing psycho-education (in 20–30 min presentations) on that week’s topic, sometimes including a video or relevant theories such as described in Table 1 (below). After this, the parents would work individually or in groups, either further exploring the chosen topic, or practising new skills in the weeks to come. Moreover, there was time and opportunity for negotiation with the parents regarding some parts of the programme. Parents were encouraged to share their experiential knowledge during all sessions, and their input was used to adjust the sessions accordingly. This approach was meant to help build the parents’ trust and self-confidence in taking an active part in the programme (Natrajan and Thomas 2002). Table 1 offers a more detailed overview of the content of each session.

**Table 1:** Overview of programme sessions

Session	Activities/explorations	Home exercise suggestions
Session 1: Creativity and Play	Mind–Body Centring exercise Getting to know each other with an improvisation game and music First exploration round: *what concerns do we have regarding the parenting of our children?* *Reflecting on feelings, thoughts and body sensations* Psycho-education on *‘play’* and the value of *‘creativity’* Group exploration: *what do you do for fun*/*quality time with your family? What excited you as a child?* Group activity: in small groups, designing together a variety of games based on given props (scarf, newspaper, etc.) Closing reflection on use of creativity and play with adolescents	Find new ways to be playful with your family, thinking about *when* (for instance during dish-washing or dinner) and *how* (following the initiative of your child, or starting something yourself) Write a few sentences on what new outcomes were imagined about the relationship and how it felt to be creative and open about the direction of the interaction between you and your child
Session 2: Self-Awareness and Bonding	Mind–Body Centring exercise Troubleshooting: how did the ‘take home’ exercise go? Drama therapy exercise for bonding & relating. ‘A dialogue with the back’, (Pendzik n.d., p. 3) Psycho-education on *Bonding*. Introduction to the Still Face experiment video (Tronick et al. 1975) Individual activity: reflecting on own childhood, ‘safe attachment’ with parents and IWM (in what ways were we nurtured vs. hurt?) Emotional Freedom Technique (EFT) to learn as a self-healing technique Closing round of reflection: *what are the ways in which you connect with your child?*	Continue to explore what happens when you connect with your child in various contexts during the week Reflect on what emotions you recognize in them, and how this makes you feel How do you usually respond? And what happens when you pay more attention to your own internal processes?
Session 3: Communication 1	Mind–Body Centring exercise Trouble-shooting and reflection on process so far Introduction to *Communication: NVC and Faber and Mazlish* Group activity around universal emotions and needs [Non Violent Communication (NVC)] Group activity with vignettes: act out scenes and break down communication patterns through role-plays. Faber and Mazlish (2004) Closing with EFT	Hand-outs on the subject of ‘emotions and needs’ (NVC). Practise by silently tuning into the emotions and needs of your child in the coming week See if you can sometimes make an educated guess about what your child might need in certain situations
Session 4: Communication 2 + Closure	Mind–Body Centring exercise Final Trouble-shooting session Continue with practising different *conflict* scenarios by incorporating creativity, bonding and communication in problem-solving behaviours. Parents bring in hypothetical *real-life conflicts* with their children, and are guided to use what they have learned to introspect, act and reflect in better ways Closure of the programme: *what have we learned and what do we look forward to?*	No take home exercises

## Method

### The Research Context

The study took place in Pune, a large and rapidly developing city in the Indian state of Maharashtra. The research team comprised mental health professionals and volunteers who had previously worked together in other projects that aimed to improve young people’s mental well-being under the umbrella of a suicide-prevention non-governmental organization (NGO) in Pune (de Wit et al. 2016). Pune has changed drastically over the course of the last 30 years due to market liberalization and rapid economic growth (Barua 2012). To recruit parents, the NGO’s project team collaborated with a private secondary school. The school’s Principal was also concerned about the stress facing parents and young children, and was willing to offer her school as the location for the parenting intervention. During a presentation at the school, parents were informed in detail about the programme. We looked at ways to frame it in a way that would be sufficiently inviting for parents to participate, deliberately avoiding words such as ‘therapy’, but emphasizing the issues that are commonly experienced in middle-class Indian society. For this reason, a video was shown, called ‘Race to Nowhere’, developed in the USA in 2010 (Trip 2010) as a response to excessive academic pressure. We particularly addressed the nature of the programme, informing them about what Natarjan et al. (2002) describe as ‘taking responsibility’ in creating change, to avoid too much reliance on the psychologist for solutions. Parents and researchers decided together that the programme would be implemented during four three-hour sessions held every Sunday, and take place in a large classroom.

### Research Methods

The parents who signed up for the programme agreed to participate in semi-structured, in-depth interviews before it started. In the pre-interviews, parents were questioned about challenges in their parenting practice, and which questions they would like to explore and gain answers to during the programme. The most commonly reported parenting questions that were abstracted from these interviews were used as input to further develop the parenting programme, as part of a PAR cycle (Regeer et al. 2009). Each semi-structured interview was conducted by independent researchers and lasted approximately 30–45 min. These researchers were volunteers from the suicide-prevention NGO who had a background in psychology and had been trained for 2 days to conduct the interviews with parents according to a common topic-list. Both the pre- and post-interview topic list included *main parental concerns, *parent–child communication, *coping strategies and *expectations from the programme. The post-interview expanded on *the experiences of parents during the programme (likes, dislikes, emotional processes and suggestions for improvement), *the lessons learned, *how these lessons were integrated into their daily lives and *in what ways they looked forward to their future parenting. The post-interviews with eight parents who participated in at least three of the four sessions lasted 45–60 min each.

It was also considered useful to capture the key lessons about the intervention itself—its success, relevance and potential weaknesses. For this purpose, the parenting sessions were recorded and transcribed verbatim. Two researchers made observations during each session, based on an observation guide. Observations about parents’ participation level, interaction patterns and attitudes were noted and used as data for this study. In addition, there were ‘debriefings’ with the facilitators and independent researchers to discuss each session. To guide these debriefings, we asked such questions as: ‘What do you feel went well?’, ‘What were the outcomes?’ and ‘What challenges did we face?’ Minutes of these debriefing sessions were then used as data for this study.

### Analysis

A qualitative, grounded theory approach was used for the analysis. As Strauss and Corbin (1998, p. 40) explain, explorative qualitative research is particularly valuable when aiming to understand the experiences of participants regarding a particular intervention. Interviews were recorded and transcribed verbatim. Some 16 pre-intervention and eight post-intervention interviews, as well as the field notes, were first read by two researchers, after which they were initially discussed. Then two researchers undertook a process of open coding to identify, name and categorize certain themes that parents found important during their participation in the programme, and in their subsequent parenting. Following this phase, the researchers developed a common coding list for selective and then axial coding (Table 2, in the "Appendix"), aiming to discover categories, sub-categories and the elements of the programme that may relate to these, along the line of their shared or related characteristics and dimensions (Strauss and Corbin 1998; Charmaz 2000). Emerging patterns were then identified that directly answered the research questions. These were then discussed with the co-authors before writing up the results.

### Ethics

Interviews were conducted, subject to the parent’s wishes, in their own home or in their children’s school. A declaration from the VU University’s Medical Ethics Committee for non-WMO (confirming that the Medical Research Involving Human Subjects Act does not apply to this particular study) was obtained in 2014 (Reference Number: 2014.170). All participants were informed about the objectives, that their participation was voluntary and their right to leave the study at any point, for which they signed informed consent forms.

## Results

Here, we first briefly reflect on the demographic backgrounds of the participating parents and the setting of the parenting programme. We then describe what the parents learnt about their questions before attending the programme, namely:

**Table:** 

A: How can we, as parents, help our children attain their academic potential, whilst shielding them from too much societal pressure, and without becoming too stressed or burdening them?
B: How can we remain better connected with our children as they grow up in this new era that is unfamiliar and sometimes daunting to us?
C: How can we help our children become independent individuals without risking physical or mental harm?

Two important findings surfaced in relation to these questions. First, we found that the exploration and practice through both the ‘creativity and play’ and ‘self-awareness and bonding’ sessions (weeks 1 and 2) helped parents to understand and do things differently in order to strengthen their connection with their children, particularly by becoming more conscious of the need for family quality time (question B). Second, we found that the ‘communication’ sessions (weeks 3 and 4), and particularly the emphasis on internal working models, positively influenced the interaction patterns between parents and children, which became more dialogical in nature (question A and C). Both findings are substantiated in depth below. In the final part, we reflect on the TOC by explaining which principles seemed to have contributed to learning among parents, and how the programme helped to reduce parenting stress in general.

### Programme Setting (Timing, Place and Gender Differences)

After the presentation in the school, some 16 parents (seven men and nine women) initially expressed interest in participating. Of these, 12 parents attended the first session, and 8 (three men and five women) eventually completed at least three if not all of the sessions. All parents had at least one or more children in the age range of 11–18 years old. Of the twelve parents, some 10 reported themselves as middle-class and two as upper-middle class, with their occupational backgrounds in business (N = 3), law (N = 1), management (N = 2), teaching (2) and engineering (N = 1). Nine of the parents held a (post)-graduate degree, while three mothers managed the household and did not continue their studies beyond college. Most parents had a good command of English (speaking and listening), although three women were less confident in English. These women were offered translation services but decided to drop out after the first session. The majority of parents signed up in couples, although three women came and stayed without their husbands. Seven of the parents lived in nuclear families, while five lived in joint households with an extended family. Finally, most participants were relatively young, aged between 33 and 45 years.

Regarding the setting of the programme, it was noticed that ‘time keeping’ was somewhat challenging. Most parents had a full weekly schedule, so attending a 3-h programme every Sunday was seen as an extra investment of time. This led to some last-minute ‘no-shows’, as well as parents sometimes arriving relatively late. Such occurrences somewhat disturbed the dynamics of the group process at times. We also saw that there was a slight overrepresentation of women in most of the sessions, and that mothers were also typically more open to being vulnerable. One of the research assistants mentioned in the debrief of session 2, for example: ‘In a way I feel we should pay more attention to the men that were there. They are more silent and find it more difficult to express, so they might need some more encouragement.’

### Quality Time: An Important Reminder

The idea of spending more quality time with children turned out to be an important take-home message for many respondents in the post-interviews, particularly because parents had previously viewed this as less important as their children grew older. One mother, who had previously said that she found it difficult to connect with her son because he did not confide in her easily, said in session 2 (1 week after the session on ‘creativity and play’):

I am not so much of a playful mum; I really don’t like to play games. But I realized now how often I actually said ‘no’ to my child’s requests and that I was missing out on something vital here. So last week he came home from school, and it was raining outside. He asked me: ‘Mum, let’s go and dance in the rain!?’ My normal response would have been: ‘No! Of course not, you will be dirty!’ But I had the exercise in my mind and actually thought to say ‘Yes’ for a change. And I even said I would go with him. His face was glowing then, and it made me feel good as well.

In the post-interview, this mother mentioned that finding a balance between saying ‘yes’ and ‘no’ had really worked for her. She now tries to say ‘yes’ at least half of the time to such playful opportunities for a quality connection. These are not just formal games but can be any time spent together. As one father mentioned after session 2:

I tried to do this thing this week, but I thought at first the whole while that I did not have time. And also, I thought that perhaps he doesn’t want to play with me anymore, since he is also getting older now. But then I just brought him to bed again for example. I hadn’t done that for a while. And it was nice, because even if it’s not playing it’s connecting in the small moments you have.

Other parents mentioned that it was helpful to work with other parents on finding different ways to create quality time with their children (through, for instance, the exercise with different props). Parents practised with these ideas and shared their experiences in the following sessions. About half of the participants mentioned that they would more often interact playfully with their children, or find more ways to spend quality time with them, particularly by using daily events, such as ‘dinner time’ or ‘doing dishes’, as opportunities. Still, this was not easy for *all* parents. One mother found it difficult to let go of the habit of focusing on more serious matters with her children. She said: ‘The thing is, my oldest is always ready to play but I hardly am, because I always refocus her on her studies. So I am aware now that I would rather do things differently, but the common reaction often comes back.’

With regard to the topic of ‘self-awareness and bonding’, and fostering the connection with adolescents in this particular generation, parents felt they had gained valuable insights during the programme which they used in their daily practice. First, for some parents, it was very revealing to become more aware of the impact of their own emotional state and responses on the parent–child connection and the children’s well-being. The programme reminded parents that children are highly receptive to their parents’ responses and accordingly make a mental note, for instance, on whether to share something that is important to them. In this ‘self-awareness and bonding’ session, parents were familiarized with the concept of ‘secure attachment’ through a video illustrating a well-known ‘still-face experiment’ by Tronick et al. (1975) in which a mother’s facial expressions lead to either comfort or confusion, and sometimes even distress, in a young child. Afterwards, parents were asked in an exercise to reflect on their experiences of how their children commonly respond to their emotional state and vice versa. During this reflection, one mother said:

For me, quite a lot of my life I have dealt with stuff like feeling low, or depression most of the time, so for me it was a big eye opener about how it really affects a child if a parent is like that. I realized that even though you are there physically but not really there, the child gets frustrated. I’ve noticed that it does make a big difference to a child if you are actually spending quality time, and that playing in any sort of way, or fooling around or anything, does make them really happy, that is for sure. I have been trying to make an effort to do that.

A father of an adolescent boy also shared his reflective learning:

What happens sometimes is that we don’t think that what we do or what we don’t do makes any difference to them. But children pick up very quickly from parents and we should be more attentive of this. So I end up turning on the TV instead of doing something fun with my children. Like when we discussed the question, ‘when was the last time you played with your child’ I remembered how long ago it was and that it would be nice to get involved with that more. So I’m more aware, and I think this affects my daily relationship with my child.

### Connection how? A Practical Learning Example

During the second (‘self-awareness and bonding’) and third (‘communication’) sessions, parents explored various ‘when’ and ‘how’ challenges that would surface in remaining connected with their children. One of the most commonly experienced challenges was children’s use of the internet. Parents would often feel fear and despair when they considered that their children spent too much time online. During the sessions, the psychologist would lead the parents through a sequence of questions to discover together what particular thought *patterns, emotions* and *needs* were underlying their instinctive responses to their children’s ‘internet use’, and how this could affect their parent–child connection. After such explorations, one father explained, for instance:

I feel that what is stopping me to react warmly is that I feel as a father I have my responsibilities and I have to do certain tasks within the time frame I am given. I think that comes from my childhood, where I was too much pressured to perform myself.

After such introspection, parents were invited to tune into their children’s emotional state while being in conflict about, for instance, their use of the internet. Role-plays were used to discover what difficulties the parents faced in interpreting the situation clearly. Afterwards, EFT was practised to heal emotional and cognitive blockages regarding the situation (Box 2).

**Table:** 

**Box 2: Illustration of Intervention: Issue of ‘internet use’ [Session 3]**
Father 1: I have a situation as well that I would like to put in the pool. There is a big challenge with internet. It’s a constant battle to try to control the time that they spend on internet and computers and to make sure they don’t get too influenced by the internet.
Mother 1: Yes, I am struggling with that as well.
Psychologist: ‘Let’s try this by acting out a role play.’
*One father and one mother played an adolescent child and a parent. The father in the role of the child would be stuck on his computer and not listen to any warnings of the parent to get off the internet. During and after the role-play, the group reflected on what happened*.
Father 1: The child is very stubborn and wants to stay on the computer, and when such things happen, we say: ‘don’t do this, don’t do that.’ We immediately go into that mode of talking. We become agitated and we get into a conversation of firing words, back and forward. So how do you handle this situation, when you’re getting agitated and you’ve got a child who maybe doesn’t react even to what you are saying? So then the frustration builds up.
Psychologist: I see, so we see that there is quite some learning to be had together because, as a parent in this, when a child usually doesn’t respond, and he is group chatting on the internet, it does something to us as a parent. And that can stimulate an emotional response within us. First, though, as a parent, could you tune into what the needs might be of that child? What do you think?
Mother 1: He just wants to chat.
Mother 3: He might have the fear to not be part of the group? To be left out?
Psychologist: Right, so you’re seeing that he’s trying to connect with other peers, which is very age-appropriate behaviour. So when we are thinking of that, what happens to our initial emotions about the situation?
Mother 1: I’m just thinking, at the beginning when a child answers to you in a certain way, you will not know how to respond to it because you would first think: ‘Hey, I would not have dared to talk like that to my parents!’ And you feel that he’s disrespecting you…

The role-play continued on from here, and participants used the NVC guidelines to tune into the child’s situation, and also to share why they had certain concerns, or regarded certain principles or parenting rules as important. The rest of the group would follow the role-play and reflect on how much the communication with their child had to do with their internal communication simultaneously. Much of this had to do with the thought of *not being in control*, or *not being good enough as a parent*.

The psychologist helped parents to conduct a process of self-healing regarding such fundamental blockages (‘I am not in control’), through performing self-administered EFT. This is a technique where stressful emotions or thoughts can be processed through a sequence of tapping with the fingertips, while pronouncing the sentence: ‘Even though I … (fill in the blanks, for instance: *I feel small when my child does not listen to me*), I still deeply and completely love and accept myself.’ Through these role-plays, parents reflected on their children’s needs as they are growing up: an increasing need to relate with their peers, the need for privacy and the need to be independent while also feeling supported. Parents returned after the third session with examples of how they used these insights in their parent–child interactions. One such example is shown in the quote by one mother below, as she is reflecting on her son:

Yesterday, I had a parent–teacher meeting. And my eldest scored very low on his maths test. And he knows what kind of mother I am, that I will go straight to the teacher to ask: ‘Why does he have such a low mark?’ But now I think my son was feeling embarrassed and afraid because he did not even go into class when his mark got declared. And the idea that I would talk to the teacher was making him very nervous. So this was the first time I actually asked my child: ‘How are you feeling, what do you want me to do?’ I tried to tune into his need for trust and safety. So, after that, I didn’t feel it would be good to seek for the answers from the teachers on why he got less marks, but we looked at it together. This made my son also feel good and more confident. He had a different expression on his face.

### Parent–Child Communication: What’s in ‘me’, Affects ‘us’

During the programme, there was a strong emphasis on parent–child communication, specifically in the third and fourth sessions. The purpose of this was to respond to the questions of parents on how to help their children become autonomous thinkers and independent actors. We hoped the sessions would help to reduce the anxiety parents experienced regarding certain objectives they had set for their children, not by neglecting the objectives, but by understanding the need behind them. Such processes can be quite revealing and offer an opportunity for parents to change their habits so that they are increasingly deliberating matters with their children as they mature rather than just trying to get things done.

For instance, parents would talk about why they found it so important to make sure their children studied hard. They indicated that it has to do with their desire to see their children safe and happy, and this is the way they strategized the ‘how’ (e.g. putting a lot of emphasis on achievements, school work and results in their daily interactions). In the sessions, parents talked about what it would mean if this need were fulfilled in other ways, and if that would bring up feelings of fear. We also looked at the potential needs of the children themselves. In role-plays, parents practised tuning into their own—as well as their children’s—needs, and how to recognize and help express these needs in their children. Some of these imaginary situations were derived from the parenting book of Faber and Mazlish (2004), including various relevant conflict scenes between parents and children. One example is a scene of an adolescent girl asking her parents if she can go on an outing with her friends (an idea which particularly fathers were against even considering at first).

**Fig. 2:**
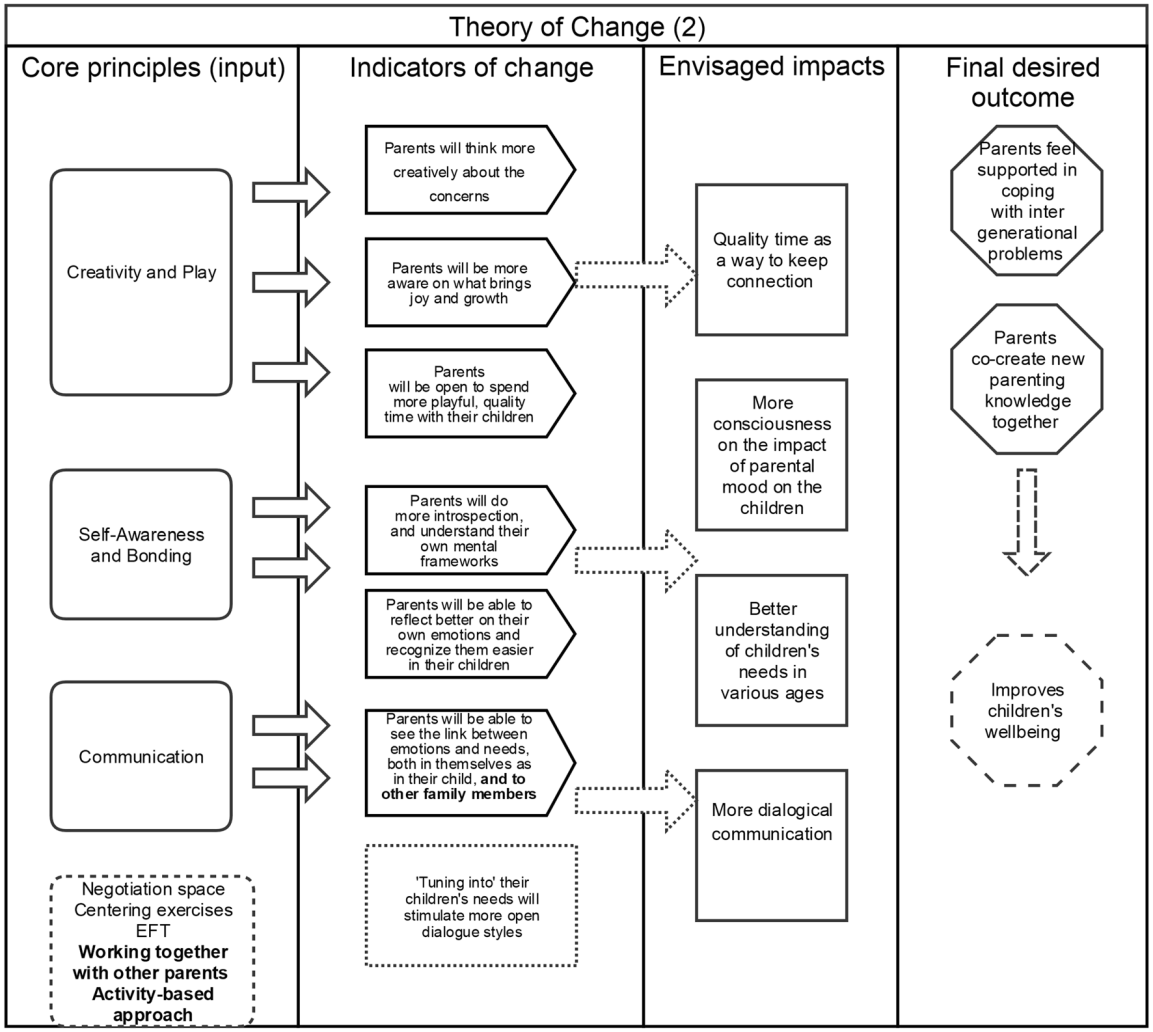
Theory of change, adapted based on results. The stars indicate the additional functions of change gained from this study

During the post-interviews with parents, all said they had learned valuable lessons with regard to connecting and communicating with themselves and their children, although the learning differed from parent to parent. In relation to parent–child communication, we perceived it as functioning on a spectrum, ranging from a closed, one-way communication style, towards a more dialogical, two-way interaction. Parents began to take various steps in the direction of more dialogical communication styles (Fig. 2). First, it seems that some parents had become more aware of the needs of their children, but still used one-way interactions with their child to get things done. This is reflected in examples of some parents who seemed to use the learning from the programme mostly to make sure that the children understood why they, as parents, make certain decisions for them. A mother explained, for instance, about her daughter’s autonomy:

There are changes in a positive way. About how I feel my daughters should manage and control themselves, how to maintain a distance to boys and why it is important. I learned to tell them why I feel worried. Now the generation is such that they feel there is nothing wrong in building relationships. So I have shared some experiences of my own with her, some other ones also that were bad, and now she understands that her mother is worried because of the risks in society. So it is good from that point of view that she understands now how to behave and what to do and what not to do.

At the other end of the spectrum, some parents discovered ways to support autonomous thinking in their children, without jeopardizing their own parenting objectives or neglecting their genuine concerns. This essentially requires parents to, at least for some time, let go of fixed objectives of what the interaction should achieve, such as: ‘by the end of the conversation, I want my son to clean up his room immediately.’ Several parents showed examples of this. One mother, who had many concerns about her daughter’s studies and used to focus on making her study, said during a post-interview:

What I have realized is that most of the time I used to have one-way communication with my child that comes from just making her do things. Now, I am trying to create space where I can spend time with her talking differently as well. Not as preacher, or having always a goal or achievement in mind. But communication that is nice in itself. Now I also see that she will do things on her own, needs to have space to decide when she wants to do these, and that we can actually give her a bit more space. We can channelize, but we can’t do it for her or force her. You can give her a plate, but she has to decide whether to eat it.

Another mother explained:

One really nice thing that I learned was about communicating, where we did a small act about how it is when someone says, ‘this happened to me’ and you say ‘oh big deal… you’ll get over it!’ Instead of doing that, if you can get down to that level and think about what it must be feeling like for the child at that time, and say ‘I understand, I know.’ No one can actually solve your problem but if they just show compassion towards it, it really makes them feel that they can do something about it themselves. That is something I really try to do.

Finally, for some parents, the programme meant that they became more aware of their own upbringing, fears and thoughts, which have a fundamental impact on parent–child interactions. This turned out to be valuable for many of the participants. As one mother explained, for instance:

For me, I was kind of going through a period of growth myself. The facilitator had said that it was also about looking into your own self. For me, that made a lot of sense because, when you’re going through that, many things come to you, of how you perceive and see everything in a clouded way. If your child comes up to you and says: “You don’t do this well”, instead of dismissing it, you actually delve deeper into it and see why it hurts you. And as these clouds start to clear, you don’t see it the same way any more. So that was one of the things; that you react to events because you’re seeing it from a perspective, which is not necessarily true. That was a great insight for me.

These parents now use these insights to empower themselves, and consequently their children. Furthermore, one mother noted that the programme not only changed communication with her children but also with her husband (Fig. 2):

It’s the way of looking that is different … I respond less aggressively to my husband now because I can shift the focus from my own frustration to what it is that I need and what he needs. It becomes clearer. Earlier I used to think that I am suffering so much from this person but I get less caught up in this way of thinking now, and it helps the communication. That leaves a nice energy for us.

### Useful Stress-Reducing Principles

We hoped that the explorations in the sessions would help parents to feel slightly better or less stressed about elements of their parenting practice. The topic of their children’s schooling was very much an integral part of all the sessions, and many practical examples were derived from parents’ experiences with their children’s education. Over time, however, parents became more relaxed in their high expectations because of their joint discussions (Fig. 2). On several occasions during the post-interviews, parents would refer to peer support as a significant contribution to ‘the whole experience and as lessening the burdens that they’d previously faced.’ Parents found it comforting to know that certain personal concerns were shared by other parents, creating a feeling of togetherness that was supportive and healing in itself. Regarding this, one mother said:

It was nice that in this group we were with parents who acknowledged that there were concerns about education and wanted to do something about it. And trying to do things differently together: that is important. To jointly see if we can be a bit more relaxed about it all.

Through learning about parenting together, new ideas and ways of handling situations were brought up. Some parents said that sharing experiences and reflections among the group members had given rise to some important eye-opening experiences and had brought them new insights (Fig. 2). As one other mother said: ‘I found it such a comfort to notice that when I did not know the answer, I could get it from someone else.’ This was witnessed in the programme as parents would often smile and sigh in recognition of each other’s stories, but also help each other get to important lessons and bring them into practice. In the third session, for example, parents shared their worries about not being able to get their children to study and tell them about what happened each day at school. When one parent raised the question of how to deal with this, an interesting discussion started, leading to the idea that the child’s ‘not listening’ was an indicator for *themselves* to start listening more and tune into their children’s need for privacy. About this, one mother said: ‘Some things we kind of know, but it’s mostly that you’re sharing it with other parents and other parents are doing the same thing, which helps to improve.’

Parents also felt it was valuable that there were many role-plays and exercises to practise with the material. One father suggested that there could be more role-plays or that children could be included if there were more sessions. One mother said:

I really liked the activity-based approach, because we can read ample things on the internet, but this way everyone was actually feeling it and working with it. This is good, because we Indians take time to open up and because of this we did open up to each other.

Finally, during the programme, if intense emotions or thoughts surfaced among participants, the psychologist would help the parents process these through self-administered EFT and ‘centring’ techniques. This helped parents to relax during the sessions, as well as in the following days. Parents, for example, would usually arrive in the mornings in a rushed state but often linger about calmly after the session was finished (Fig. 2). Each session started with a centring exercise, to which parents responded as a helpful and supportive element. For some parents, these exercises were not new as meditation and mindfulness are common in much of Indian culture but they were no longer practising them as much. Now, these exercises were helping parents to relax and be more fully attentive to the situation, which according to their self-reports reduced the stress in their parenting experience.

## Discussion

It is notoriously difficult to measure the impact of an intervention on the behavioural changes of participants, as these are embedded in social contexts and dependent on various interacting social conditions (Breuer et al. 2016). As such it is advisable to evaluate the mechanisms through which interventions might work, or ‘the resources a program offers to enable their subjects to make them work’ (Pawson and Tilley 2004, p. 6), before testing the long-term effects in a controlled setting. In this article we reflected upon participants’ learning experiences and how they perceived the Creative Stress-Relieving Programme. First, we noticed that most parents gained valuable lessons regarding the importance of quality time, play and creativity with their children. This is a significant change in a context where there is little time for play or free exploration in the schedules of many Indian children, and parental attention is largely focused on studies (Deb et al. 2010, 2015; Natrajan et and Thomas 2002). For some parents, it was slightly unusual but also a relief to act playfully, make up games, and connect with others (parents in the programme, as well as their children at home) in an unanticipated way. Deb et al. (2015) describe how Indian parents often validate their existence by making sure their children are successful, leading them to repeatedly encourage children to study, at times even removing televisions, opportunities for socializing, or any other forms of entertainment from their children’s daily life. Competition, high expectations and ‘over-pressurizing’ are acknowledged as a growing problem in Indian middle-class families (Natrajan and Thomas 2002). The focus on play and creativity in this programme allowed parents to re-evaluate their developmental goals, as well as the value of quality time they spend with their children, apart from the daily chores and other responsibilities. Most parents felt they found a new balance in relating to their children and more often said ‘yes’ to opportunities for quality time, making themselves and the children happier and more relaxed.

Parents reported other lessons about ‘communication for autonomy’. Indian culture is often characterized as more collective and hierarchical or vertical, promoting social cohesion and interdependence, rather than autonomy (Baptiste 2005). However, such dynamics are changing quickly (Chadda and Deb 2013; Singh and Bhayana 2014). In middle-class families, conflicts between parents and children increasingly occur as a result of the clash between traditional culture and the increasingly individualistic nature of modern society (Natrajan and Thomas 2002). The parent–child communication is understood here as a spectrum from ‘closed communication’, which fits the demands of traditional society, to more ‘dialogical, open communication’ under the umbrella of ‘stimulating autonomy and independence’ in children (Chadda and Deb 2013). In our study, some parents took various steps towards more dialogical communication styles and learned to disclose their own *thoughts, fears* and *feelings* underlying certain behaviours. Some parents also made efforts to empathize with their children’s context and practised allowing more negotiation. They learned from the programme to reflect on the underlying reasons for their fears, the needs they prioritize, and the strategies they usually use to protect these needs with regard to their children. The NVC exercises also stimulated them to connect to the needs their children may have in different stages of their life. For some parents, this improved their relationship with not only their child but also their partners because they responded less impulsively to stressful events. Although not many interventions for parents, including NVC training, have been studied with regard to their effects, programmes with other target groups have shown that NVC can improve empathy (Nosek et al. 2014), increase emotional verbalization and reduce feelings of distress (Wacker and Dziobek 2016) and, ultimately, improve interpersonal relationships (Marlow et al. 2011). The fact that parents slightly changed their attitudes and communication styles in response to this programme shows that, in relatively few sessions, the connection between parents and children, as well as between parents, can be improved. Through the qualitative data was found that some parents felt the programme had also stimulated an improvement in the relationship with their partner. Such improvements could potentially help to further reduce parenting stress, as was found in other studies (Lionetti et al. 2015). On the whole, such interventions are rarely described in the context of inter-generational challenges, but endorsed by other scholars who state the need to pay more attention to efforts to influence family practices (Sonawat 2001; Singh and Bhayana 2014).

But there were also parental questions that were less resolved, according to data from the post-interviews. Parents still expressed difficulties in dealing with the societal pressures on their children to be educated in a rigid and competitive school environment, knowing that this will only increase when their children enter the job market. This is a genuine fear most parents experience and continue to foster when sharing their children’s successes, shaming failure and comparing each other’s progress, nurturing and maintaining a culture of competition rather than a sense of playful learning in society (Deb et al. 2010; Rao 2009). At the same time, we see that things are changing slowly, and that more parents accept their children studying subjects such as the arts and psychology, and not just engineering or medicine. The programme helped parents to explore jointly and build a different perspective on their communal culture, hence forming a stronger stance against such societal tendencies. Such processes are often witnessed in PAR approaches (Lewin 1946; Kemmis and McTaggart 1988; Ferrance 2000), which focus on stimulating both personal and social development in the group setting, creating theme discussions that touch upon all facets of society (Bergold and Thomas 2012). Bergold and Thomas (2012, p. 1) describe this positively reaffirmed aspect of such participative group processes in PAR as:

The participatory research process enables co-researchers [participants in the study] to step back cognitively from familiar routines, forms of interaction, and power relationships in order to fundamentally question and rethink established interpretations of situations and strategies.

Finally, we reflect on the *theory of change* to understand if, and how, this parenting programme helped parents. Quite remarkably, after only four sessions, parents did say that they felt calmer, mostly through having recognized that other parents are not only experiencing similar dilemmas but that they also share an interest in changing certain patterns in their parenting practice. The strength of using a *theory of change* in this context lies in the fact that we could evaluate *why, how* and for *whom* the programme such results came about (Connell and Kubisch 1998), which could be useful for programme designers and facilitators aiming to foster similar results in other contexts. We saw here that the *activity-based approach*, including a wide range of role-plays, interactive games and discussions, helped to create a safe space for parents and induce a genuine culture of change. We would also argue that the *accessibility* and *feasibility* of this programme was key here, even though there were some struggles in implementing this programme, and doubts remain as to whether it managed to reach the right parents. Participants may have dropped out as a result of their beliefs regarding family therapy, as well as the role of a counsellor (Ingoldsby 2010; Natrajan and Thomas 2002). As Chadda and Deb (2013, p. 305) sensibly argue in their article on the use of family therapy approaches in India, the therapist needs to be aware of and considerate towards the cultural issues that may arise, particularly when hoping to successfully bridge inter-generational gaps. Chadda and Deb (2013, p. 305), for example, explain: ‘Directive approaches might be more suitable for traditional families, as the therapist is often looked upon as charismatic, authoritarian and in control of the session.’

Here, we found that some parents expected more directive guidance from the facilitator, and showed some confusion and resistance as a result of the interactive character of the programme. Some parents may even have decided not to participate for this reason alone. Natrajan and Thomas (2002, p. 500) explain that Indian parents often think of therapy ‘as a process where they were active participants responsible for their own healing.’ This is something to consider when designing future programmes for parents who have particular difficulties in negotiating modern issues with their children, as they are also most likely to drop out (Ingoldsby 2010). However, we would still argue for a balance between bringing in more directive approaches and encouraging parents to engage actively in re-creating their parenting practice through a process of self-reflection, questioning and experimentation, especially for this generation of parents.

## Limitations and Final Considerations

To our knowledge, this is the first study to report on a programme that deals with inter-generational challenges experienced by middle-class parents in India (or other rapidly developing countries). This study thus provides new perspectives on how to engage such a target group in exploring their parenting questions in an interactive setting, thereby reducing parenting stress (and in turn improving the well-being of their children). Furthermore, a TOC perspective on programme design, such as employed in this study, could help other professionals to make their assumptions explicit, which could support the implementation and impact of similar programmes elsewhere (Breuer et al. 2016). There were, however, some limitations to this qualitative study, as we did not measure (long-term) outcomes, such as parenting styles, communication patterns or parenting stress, or mental health-related outcomes in children. We hope that in the future more rigorous studies (including real-life observations and validated scales, for example) could be undertaken to validate the self-reported changes found in the research. This study does, however, show that it is feasible to fill the mental health gap for middle-class families by implementing low-threshold, culturally sensitive programmes through community efforts and school networks. We encourage other mental health professionals to bring together the basic resources required to develop and study similar interventions for families, in order to improve parent–child relationships in other parts of the world that are undergoing rapid change.

## Appendix

See Table 2.

**Table 2:** Overview of the coding scheme

Merging and recurrent topics (Patterns), regarding;	Subcategories	Categories	Interview illustrative quotes
1. Learning about parenting question A (academic goals)	Identification of underlying fears Reflecting on the importance of ‘letting go’ Showing examples of thinking creatively about future goals	Feeling less stressed about one’s academic goals Awareness of the individuality of the child	…
2. Learning about parenting question B (connection)	Importance of saying ‘yes’ to play more often Examples of reflecting on one’s own internal working models Showing knowledge on the impact of one’s emotions on the child Examples of ‘tweaking’ patterns of listening and communication	Importance of ‘quality’ time Importance of communication	…
3. Learning about parenting question C (autonomy)	Awareness of old and new ways of communication Identification of children’s needs Finding ways to provide more autonomy Showing examples of finding the balance in dialogical communication	Expressing the importance of listening Expressing the importance of expression	…
4. Patterns that reduced parenting stress (instrumental factors)	Sharing/working with other parents Mindfulness & EFT Activity based approach	Co-creating knowledge	…
5. Patterns responsible for the overall feasibility of the programme	Time constraints Expectations Children not involved	Reaching out to parents in need, at the correct time	…

## Footnotes

1 In India there is a trend of complementing formal education with private tuition in order to support and improve children’s academic development (Sujatha 2006).
